# Clinical significance of the systemic immune-inflammation index in relation to pathology, treatment and outcomes in acute appendicitis: a retrospective study

**DOI:** 10.1080/07853890.2025.2585543

**Published:** 2025-11-18

**Authors:** Lerong Yan, Zhenhai Chen, Xia Xiao, Zhongfu Li, Yongguo Liu, Wei Zhao, Beige Zong

**Affiliations:** ^a^ Department of General Surgery, Chongqing Emergency Medical Center, Chongqing, China; ^b^ Department of Hepatobiliary Surgery, Chongqing Emergency Medical Center, Chongqing, China

**Keywords:** Acute appendicitis, systemic immune-inflammation index, drainage tube placement, hospitalization outcomes

## Abstract

**Introduction:**

Acute appendicitis is a common abdominal emergency. Systemic Immune-Inflammation Index (SII) is a biomarker reflecting immune status and inflammatory response, but its role in acute appendicitis remains poorly characterized. This study aims to investigate the potential diagnostic and therapeutic value of SII for assessing infection severity and prognosis in acute appendicitis.

**Materials and methods:**

This retrospective study analyzed clinical data from patients with acute appendicitis admitted to our hospital. The SII was the primary exposure variable and calculated as SII = platelet count × neutrophil count /lymphocyte count. Outcome variables included length of stay and total hospitalization costs. Covariates included age, sex, admission time, body temperature, shock status, comorbidities, hemoglobin, procalcitonin, presence of fecalith on computed tomography (CT), placement of intraoperative drainage tubes, and postoperative pathological classification.

**Results:**

The study included 2,458 patients with acute appendicitis. Surgically treated patients had significantly higher SII than conservatively managed patients (2077.76 vs. 1526.80). SII was positively correlated with the pathological severity of acute appendicitis (*p* < 0.001). Patients with gangrenous appendicitis had a significantly higher SII values [median 2452.10 (IQR 1486.70–3920.70)] than those with simple inflamed appendicitis [median 1026.14 (IQR 505.86–1501.76)]. Moreover, SII was significantly associated with intraoperative drainage tube placement (*p* = 0.002); patients in the drainage tube group had higher SII values [median 2263.32 (IQR 1326.86–3682.69)] than those in the non-drainage tube group [median 1977.62 (IQR 1121.81–3409.83)].

**Conclusions:**

SII is a valuable diagnostic tool in patients with acute appendicitis, offering a quantitative basis for clinical decision-making.

## 1. Introduction

Acute appendicitis is among the most common abdominal emergencies, progressing rapidly and affecting individuals of all ages [1]. The 'gold standard' treatment is appendectomy, a surgical removal of the appendix that is generally regarded as routine and safe [2,3]. Acute appendicitis is also a leading cause of emergency surgical procedures worldwide [4]. It is classified into four main types: simple inflamed, suppurative and gangrenous and perforated appendicitis. Prompt surgical intervention is generally associated with favourable outcomes. However, factors such as advanced age, comorbid conditions and limited disease awareness often lead to delayed presentation, resulting in gangrenous or perforated appendicitis and, in some cases, systemic infection [5,6]. In recent years, the growing elderly population and the rising burden of healthcare costs among older adults have become major public health concerns [7]. Population aging significantly expands the demand for medical resources. Nevertheless, the current adjustments made to medical resources in China have not kept pace with this demographic change [8].

Laboratory examination plays an important role in assisting clinicians with diagnosis and prognosis assessment. However, in the past, evaluation of disease progression, treatment preference and decisions regarding intraoperative drainage placement relied primarily on clinical history, physical examination, surgical experience and postoperative pathological findings [3]. Therefore, identifying a reliable biological indicator is essential to guide surgical decision-making. Since acute appendicitis triggers an acute inflammation, related inflammatory markers include C-reactive protein (CRP), procalcitonin (PCT, ng/mL), erythrocyte sedimentation rate, interleukin-6, systemic immune-inflammation index (SII) and systemic inflammatory response index. Among these indicators, the SII is a biochemical marker that reflects immune status and the degree of inflammatory response in the body. It is calculated as follows: SII = platelet count (Plt) × neutrophil count (Neu)/lymphocyte count (Lym). SII can be used to assess infection severity and predict clinical outcomes [9,10]. A recent systematic review and meta-analysis shows that SII demonstrates strong diagnostic performance for acute appendicitis and effectively distinguishes uncomplicated from complicated cases in adults and children [11]. Another prospective, non-randomized observational study involving 151 patients developed the Biomarkers for the Diagnosis of Appendicitis in Pediatrics (BIDIAP index), which included three variables: appendiceal diameter ≥ 6.9 mm, SII ≥ 890 and the presence of peritoneal irritation. The study shows that the BIDIAP index is a simple, low-cost diagnostic tool with excellent accuracy for paediatric acute appendicitis [12]. Guo et al. retrospectively analysed the clinical data of 104 children diagnosed with acute appendicitis. The results demonstrate that integrating the SII with paediatric appendicitis score significantly improved the prediction of disease severity and surgical outcomes [13]. However, no reliable biomarker currently exists to accurately predict the occurrence or progression of acute appendicitis. Moreover, the diagnostic and prognostic value of SII in this context remains poorly characterized.

Therefore, this study aims to explore the association between SII, infection severity and prognosis in acute appendicitis. The study could provide evidence-based guidance for its diagnosis and treatment from a health economics perspective.

## 2. Materials and methods

### 2.1. Study population

This study retrospectively collected clinical data–including demographic characteristics, laboratory and imaging results, and postoperative pathological findings–from 2458 patients diagnosed with appendicitis who met the inclusion criteria and were admitted to our hospital between May 2016 and September 2024.

The inclusion criteria were as follows: (1) a confirmed diagnosis of acute appendicitis based on the medical history, clinical symptoms, physical examination, laboratory and imaging findings, and postoperative pathology; (2) receipt of surgical treatment (open or laparoscopic appendectomy) or conservative management; and (3) absence of any diagnosed mental disorder.

The exclusion criteria were: (1) incomplete clinical data for Plt, Neu or Lym counts; (2) postoperative pathological findings indicating malignant tumours; and (3) cases deemed inappropriate for inclusion by the research team.

This retrospective study utilized only routine examination results and hospitalization records of patients previously admitted to our hospital. It did not impose any financial burden on the patients, influence their clinical management, or result in any additional interventions or other ethical violations. Therefore, the Ethics Committee of Chongqing Emergency Medical Center approved this study and granted an exemption from informed consent. All patient data were anonymized and coded to ensure complete confidentiality and prevent the disclosure of personal identities. While this study involved private health and lifestyle information, the research team strictly adhered to the principles of the 'Declaration of Helsinki' and the 'International Ethical Guidelines for Biomedical Research Involving Human Subjects,' jointly issued by the Council for International Organizations of Medical Sciences (CIOMS) and the World Health Organization (WHO). All patient medical records were securely preserved, and no personally identifiable information will be disclosed in any public presentation or the findings of this study. Every effort was made to protect the privacy of the medical, health and lifestyle data of the participants within the scope permitted by law. This research was reviewed and approved by the Ethics Committee of our hospital (Approval No. [71], 2025).

### 2.2. Study variables

Patient data–including age, sex, admission time, length of stay (LOS), vital signs, complete blood count results, presence of fecaliths on computed tomography (CT), treatment methods, intraoperative drainage tube use, postoperative pathological findings, discharge diagnosis and total hospitalization cost–were collected from the medical record database of our hospital. The SII was the primary exposure variable calculated as SII = Plt × Neu/Lym. Plt, Neu and Lym were measured using automated haematology analysers and expressed in units of ×10^9^ cells/L. LOS and total hospitalization costs were defined as outcome variables representing health economic indicators. Covariates included age, sex, admission date, body temperature, presence of shock, comorbidities, haemoglobin (HGB, g/L), PCT (ng/mL), presence of fecalith on CT (yes/no), intraoperative drainage tube placement (yes/no) and postoperative pathological findings. Age was classified as advanced or non-advanced based on a fixed threshold of 80 years old (y/o) or the median age of individuals aged ≥ 60 y/o (68 y/o). Admission time was divided between 1:00 and 8:00 am or during other hours. Body temperature was classified as normal (<37.3° C), low-grade (37.3–38.0° C), moderate (38.1–39.0° C), or high fever (≥39.1° C). The shock index (SI) was calculated as SI = heart rate/systolic blood pressure. Shock was defined as a SI ≥ 1, and a normal state as SI < 1. Comorbidities were classified into chronic diseases–including hypertension, diabetes, coronary heart disease (CHD) and chronic obstructive pulmonary disease (COPD)–other diseases, and pregnancy. Hypertension was defined as a prior diagnosis by a physician and ongoing treatment with antihypertensive medications. Diabetes mellitus was defined as a physician-diagnosed condition managed with hypoglycaemic agents or insulin. CHD was defined as a physician-diagnosed condition managed with oral anticoagulants therapy. COPD was defined as a physician-diagnosed condition managed with bronchodilators or glucocorticoids. Postoperative pathological findings were classified as simple inflamed appendicitis, suppurative appendicitis, gangrenous appendicitis with or without perforation, or acute exacerbation of chronic appendicitis.

### 2.3. Statistical analysis

Statistical analyses were conducted using SPSS version 27.0 (IBM Corp., Armonk, NY). Normality tests were conducted for all variables. A *p* value > 0.05 was considered indicative of a normal distribution. Continuous variables following a normal distribution were expressed as the mean ± standard deviation (SD) ($\bar{x}\pm s$), whereas non-normally distributed variables were expressed as median (*M* [*P*_25_, *P*_75_]). Categorical variables were expressed as counts (*n*) and percentages (%). Normally distributed continuous variables were compared using the independent samples *t*-test, while non-normally distributed variables were analysed using the Mann–Whitney'hi*U*-test or Kruskal–Wallis test. Categorical variables were compared using the Chi-square test or Fisher's exact test, as appropriate. Multivariable logistic regression analysis was conducted to evaluate the association between the SII and intraoperative drainage tube placement, yielding beta coefficients and 95% confidence intervals (CIs). Missing values were imputed using the mean for continuous variables and the mode for categorical variables. A *p* value of < 0.05 was considered statistically significant.

## 3. Results

### 3.1. Relationship between SII and treatment methods

Table 1 shows the significant differences in sex, advanced age, body temperature, comorbidities, HGB, PCT and presence of fecaliths on CT among participants who received different treatment modalities. A higher proportion of male patients underwent surgical treatment, while more female patients received conservative management (*p* < 0.006). While no difference was observed in age (*p* = 0.379), the proportion of participants aged > 80 years who received conservative treatment was significantly larger than patients < 80 years. Overall, baseline body temperature was higher in participants who underwent surgical treatment than in those who received conservative management (*p* < 0.001). The proportion of patients with comorbidities–such as hypertension, diabetes, CHD, COPD or pregnancy–was higher in the conservative treatment group than in the surgical treatment group (*p* < 0.001). These findings suggest that older female patients with multiple chronic comorbidities or who are pregnant are more likely to receive conservative management, potentially owing to concerns about surgical risks. However, based on the progression trends of pathological classifications and patterns observed in clinical practice, it can be inferred that both patient groups experienced more rapid disease progression and were more susceptible to developing anaemia. While patients with acute exacerbation of chronic appendicitis may initially respond to conservative treatment and subsequently require surgery if these measures fail, outcome among the 38 elderly patients who received conservative management were likely poor, with a high risk of mortality. After repeated episodes of chronic appendicitis, patients may ultimately require surgical intervention. While in Plt counts did not differ significantly between groups (*p* = 0.22), higher Neu counts and lower Lym counts in the surgical group contributed to higher SII levels. Based on SII values (median 1526.80 (IQR 843.98–2672.67)) among patients who receiving conservative management, those with an SII ≤ 1500 may be suitable candidates for non-surgical treatment. This threshold could assist surgeons in making rapid clinical assessments. Furthermore, LOS was significantly longer in the surgical group, leading to higher total hospitalization costs.

**Table 1.** Differences between the groups of several treatment methods.

	Surgical treatment	Conservative treatment	*p* Value
Sex (*n* (%))			
Male	953 (50.8%)	257 (44.2%)	0.006
Female	924 (49.2%)	324 (55.8%)	
Age (y/o)	41.00 (29.00, 58.00)	42.00 (29.00, 60.00)	0.379
Advanced age (≥80 y/o)	58 (3.1%)	38 (6.5%)	<0.001
Non-advanced age (<80 y/o)	1819 (96.9%)	543 (93.5%)	
Admission time			
1:00–8:00 am	420 (22.4%)	121 (20.8%)	0.424
Other time	1455 (77.6%)	460 (79.2%)	
Body temperature (*n* (%))			
Normal	1401 (75.7%)	470 (81.0%)	0.002
Low fever	320 (17.3%)	91 (15.7%)	
Moderate fever	106 (5.7%)	12 (2.1%)	
High fever	23 (1.2%)	7 (1.2%)	
Shock (*n* (%))			
Yes	1732 (93.6%)	548 (94.5%)	0.452
No	118 (6.4%)	32 (5.5%)	
Comorbidities (*n* (%))			
Hypertension, diabetes, CHD and COPD	272 (14.5%)	124 (21.3%)	<0.001
Other diseases	1151 (61.3%)	383 (65.9%)	
Pregnancy	28 (1.5%)	42 (7.2%)	
Plt (×10^9^ cells/L)	196.00 (157.00, 236.00)	200.00 (158.50, 244.00)	0.22
Neu (×10^9^ cells/L)	10.79 (8.02, 13.59)	9.20 (6.31, 11.99)	<0.001
Lym ×10^9^ cells/L)	1.00 (0.66, 1.49)	1.15 (0.76, 1.61)	<0.001
SII	2077.76 (1174.48, 3456.03)	1526.80 (843.98, 2672.67)	<0.001
HGB (g/L)	136.00 (124.00, 147.00)	130.00 (118.00, 142.00)	<0.001
PCT (ng/mL)	0.13 (0.06, 0.96)	0.10 (0.06, 0.50)	0.031
Fecalith discovered on CT (*n* (%))			
Yes	142 (7.6%)	87 (15.0%)	<0.001
No	1735 (92.4%)	494 (85.0%)	
LOS (days)	6 (5, 8)	4 (2, 6)	<0.001
Total hospitalization costs (¥)	17111.21 (14383.13, 21060.35)	5417.77 (3735.94, 7683.44)	<0.001

CHD: coronary heart disease; COPD: chronic obstructive pulmonary disease; SII: systemic immune-inflammation index; LOS: length of stay; CT: computed tomography; Plt: platelet; Neu: neutrophil; Lym: lymphocyte; HGB: haemoglobin; PCT: procalcitonin.

### 3.2. Relationship between systemic immune-inflammation index and postoperative pathological classification

No significant differences were observed in sex, age (including advanced age), admission time, body temperature, shock status, comorbidities, intraoperative drainage tube placement, PCT or presence of fecaliths on CT across participants with different postoperative pathological classifications (Table 2). A higher proportion of female patients had simple inflamed appendicitis or acute exacerbation of chronic appendicitis (*p* = 0.002). Patients with simple inflamed appendicitis were primarily younger, whereas those with suppurative appendicitis, gangrenous appendicitis with or without perforation, and acute exacerbation of chronic appendicitis were predominantly middle-aged (*p* < 0.001). Additionally, the proportion of geriatric patients (≥68 y/o) with gangrenous appendicitis (with or without perforation) or acute exacerbation of chronic appendicitis was higher than that in other groups (*p* < 0.001). Among patients admitted between 1:00 and 8:00 am, the proportion with simple inflamed, suppurative or gangrenous appendicitis (with or without perforation) was higher than that with acute exacerbation of chronic appendicitis (*p* = 0.028). Because acute exacerbation of chronic appendicitis involves multiple recurrences, these patients were already aware of their condition and recognized a recurrence when abdominal pain reappeared. Therefore, more patients with acute exacerbation of chronic appendicitis were admitted during daytime hours than those with other pathological classifications. As disease severity increased–reflected in the postoperative pathological classification–the proportions of patients with low-grade (37.3–38.0 °C), moderate (38.1–39.0 °C), and high fever (≥39.1 °C) also increased, indicating an upward trend in body temperature with worsening disease (*p* < 0.001). At admission, 9.1% of patients with gangrenous appendicitis, with or without perforation, were in shock, a significantly higher proportion than that in other groups (*p* < 0.001). The prevalence of comorbidities such as hypertension, diabetes mellitus, CHD or COPD was higher among patients with acute exacerbation of chronic appendicitis than among those in other groups, whereas pregnancy was more common among patients with simple inflamed appendicitis than among those in other groups (*p* = 0.002). Further, intraoperative drainage tube placement was significantly higher in the gangrenous appendicitis group, with or without perforation, than in the other groups (*p* < 0.001). Fecaliths were detected on CT in 15.7% of patients with acute exacerbation of chronic appendicitis, a significantly higher proportion than that in the other groups (*p* < 0.001).

**Table 2.** Differences among the patients with postoperative pathological classification.

	Simple inflamed appendicitis	Suppurative appendicitis	Gangrenous appendicitis with/without perforation	Acute exacerbation of chronic appendicitis	*p* Value
Sex (*n* (%))					
Male	19 (38.0%)	414 (50.1%)	492 (53.9%)	31 (34.8%)	0.002
Female	31 (62.0%)	413 (49.9%)	421 (46.1%)	58 (65.2%)	
Age (y/o)	31.50 (24.00, 42.25)	38.00 (29.00, 54.00)	47.00 (30.00, 60.00)	42.00 (28.00, 63.50)	<0.001
Advanced age (≥68 y/o)	0 (0.0%)	75 (9.1%)	130 (14.2%)	13 (14.6%)	<0.001
Non-advanced age (<68 y/o)	50 (100.0%)	752 (90.9%)	783 (85.8%)	76 (85.4%)	
Admission time					
1:00–8:00 am	11 (22.0%)	199 (24.1%)	201 (22.0%)	9 (10.1%)	0.028
Other time	39 (78.0%)	626 (75.9%)	712 (78.0%)	80 (89.9%)	
Body temperature (*n* (%))					
Normal	48 (98.0%)	666 (81.6%)	617 (68.4%)	70 (82.4%)	<0.001
Low fever	1 (2.0%)	116 (14.2%)	190 (21.1%)	14 (16.5%)	
Moderate fever	0 (0.0%)	30 (3.7%)	76 (8.4%)	1 (1.2%)	
High fever	0 (0.0%)	4 (0.5%)	19 (2.1%)	0 (0.0%)	
Shock (*n* (%))					
Yes	1 (2.0%)	30 (3.7%)	82 (9.1%)	5 (5.9%)	<0.001
No	48 (98.0%)	786 (96.3%)	820 (90.9%)	80 (94.1%)	
Comorbidities (*n* (%))					
Hypertension, diabetes, CHD and COPD	5 (8.9%)	103 (11.4%)	144 (13.9%)	20 (18.9%)	0.002
Other diseases	32 (57.1%)	472 (52.1%)	595 (57.3%)	53 (50.0%)	
Pregnancy	3 (5.4%)	9 (1.0%)	16 (1.5%)	0 (0.0%)	
Whether drainage tubes were placed during operations (*n* (%))					
Yes	6 (12.0%)	170 (20.6%)	357 (39.1%)	16 (18.0%)	<0.001
No	44 (88.0%)	657 (79.4%)	556 (60.9%)	73 (82.0%)	
Plt (×10^9^ cells/L)	203.00 (168.75, 246.25)	203.00 (162.00, 242.00)	190.00 (149.00, 229.00)	206.00 (171.00, 244.00)	<0.001
Neu (×10^9^ cells/L)	7.18 (4.39, 9.50)	10.61 (7.66, 13.13)	11.41 (9.01, 14.42)	6.30 (3.95, 9.80)	<0.001
Lym (×10^9^ cells/L)	1.47 (0.99, 1.85)	1.09 (0.71, 1.56)	0.87 (0.57, 1.30)	1.44 (1.03, 1.78)	<0.001
SII	1026.14 (505.86, 1501.76)	1919.77 (1041.67, 3229.81)	2452.10 (1486.70, 3920.70)	893.24 (469.39, 1714.12)	<0.001
HGB (g/L)	135.00 (125.50, 147.25)	137.00 (125.00, 147.00)	136.00 (123.00, 148.00)	132.00 (123.00, 142.00)	0.282
PCT (ng/mL)	0.04 (0.04, 0.10)	0.09 (0.04, 0.31)	0.23 (0.08, 2.96)	0.09 (0.04, 0.53)	<0.001
Fecalith discovered on CT (*n* (%))					
Yes	5 (10.0%)	51 (6.2%)	73 (8.0%)	14 (15.7%)	<0.001
No	45 (90.0%)	776 (93.8%)	840 (92.0%)	75 (84.3%)	
LOS (days)	5 (4, 6)	5 (4, 7)	7 (5, 9)	6 (5, 8)	<0.001
Total hospitalization costs (¥)	14257.94 (12675.35, 17018.01)	16113.25 (13753.72, 19000.10)	18576.33 (15539.04, 23643.35)	16642.52 (13577.33, 19593.86)	<0.001

CHD: coronary heart disease; COPD: chronic obstructive pulmonary disease; SII: systemic immune-inflammation index; LOS: length of stay; CT: computed tomography; Plt: platelet; Neu: neutrophil; Lym: lymphocyte; HGB: haemoglobin; PCT: procalcitonin.

Pairwise comparisons were conducted for measurement variables that exhibited statistically significant differences across groups (Supplementary Table 1). The age of patients with simple inflamed appendicitis (median 31.50 (IQR 24.00–42.25) y/o) differed significantly from that of patients in the other groups (*p* < 0.001). A significant age difference was observed between patients with suppurative appendicitis (median 38.00 (IQR 29.00–54.00) y/o)) and those with gangrenous appendicitis with or without perforation (median 47.00 (IQR 30.00–60.00) y/o; *p* < 0.001). The Plt count of patients with gangrenous appendicitis with or without perforation (median 190.00 (IQR 149.00–229.00) × 10^9^ cells/L) was determined. This value differed significantly from that of patients with suppurative appendicitis (median 203.00 (IQR 162.00–242.00) × 10^9^ cells/L; *p* < 0.001) and those with acute exacerbation of chronic appendicitis (median 206.00 (IQR 171.00–244.00) × 10^9^ cells/L; *p* = 0.011). Furthermore, excluding patients with simple inflamed appendicitis and those experiencing acute exacerbation of chronic appendicitis, the Neu and Lym counts exhibited statistically significant differences among the remaining groups (*p* < 0.001). The SII in each group displayed a statistical trend consistent with that observed for Neu and Lym counts. A PCT level ≥2 ng/mL is commonly indicative of sepsis or even shock. Considering the PCT level observed in patients with gangrenous appendicitis in this study (median 0.23 (IQR 0.08–2.96) ng/mL), a pathological diagnosis of gangrenous appendicitis may be suggested when the PCT exceeds 2 ng/mL. No significant difference in LOS was observed between patients with simple inflamed appendicitis (median 5 (IQR 4–6) days) and those with suppurative appendicitis (median 5 (IQR 4–7) days). However, both groups exhibited a shorter LOS than that of patients with gangrenous appendicitis with or without perforation (median 7 (IQR 5–9) days) and those with acute exacerbation of chronic appendicitis (median 6 (IQR 5–8) days). Additionally, patients with gangrenous appendicitis, with or without perforation, exhibited a longer LOS, which was associated with significantly higher total hospitalization costs than those in the other groups (*p* < 0.001).

### 3.3. Relationship between systemic immune-inflammation index and intraoperative placement of drainage tubes

To further examine the relationship between the SII and intraoperative placement of drainage tubes, the data were reorganized and subjected to statistical analyses (Table 3). Among patients who underwent surgical procedures involving the placement of drainage tubes, the proportion of males was significantly higher than that of females (*p* = 0.045). The mean age of patients who received drainage tubes (median 51.00 (IQR 35.00–64.00) y/o) was significantly higher than that of patients without drainage tubes (median 38.00 (IQR 28.00–55.00) y/o). Additionally, the proportion of geriatric patients (≥68 y/o) with drainage tubes was also higher (*p* < 0.001). A significantly higher proportion of patients with high fever (≥39.1 °C) underwent intraoperative drainage tube placement (*p* = 0.012). A higher proportion of patients with comorbidities (including hypertension, diabetes, CHD, COPD and other diseases) underwent drainage tube placement, whereas fewer pregnant patients underwent drainage tube placement (*p* < 0.001). Except for patients with suppurative appendicitis, all other groups exhibited a significantly higher proportion of patients without drainage tubes (*p* < 0.001).

**Table 3.** Differences between the groups with or without drainage tubes.

	Drainage tubes were placed during operations	Drainage tubes were not placed during operations	*p* Value
Sex (*n* (%))			
Male	345 (53.8%)	719 (49.1%)	0.045
Female	296 (46.2%)	746 (50.9%)	
Age (y/o)	51.00 (35.00, 64.00)	38.00 (28.00, 55.00)	<0.001
Advanced age (≥68 y/o)	147 (22.9%)	164 (11.2%)	<0.001
Non-advanced age (<68 y/o)	494 (77.1%)	1301 (88.8%)	
Admission time			
1:00–8:00 am	142 (22.2%)	323 (22.1%)	0.970
Other time	499 (77.8%)	1140 (77.9%)	
Body temperature (*n* (%))			
Normal	470 (74.6%)	1104 (76.4%)	0.012
Low fever	106 (16.8%)	253 (17.5%)	
Moderate fever	40 (6.3%)	79 (5.5%)	
High fever	14 (2.2%)	9 (0.6%)	
Shock (*n* (%))			
Yes	45 (7.1%)	88 (6.1%)	0.368
No	585 (92.9%)	1357 (93.9%)	
Comorbidities (*n* (%))			
Hypertension, diabetes, CHD and COPD	118 (18.4%)	156 (10.7%)	<0.001
Other diseases	386 (60.2%)	759 (51.8%)	
Pregnancy	3 (0.5%)	25 (1.7%)	
Plt (×10^9^ cells/L)	194.00 (155.00, 233.75)	196.50 (158.00, 237.00)	0.563
Neu (×10^9^ cells/L)	10.72 (8.23, 13.70)	10.78 (7.85, 13.47)	0.338
Lym (×10^9^ cells/L)	0.91 (0.60, 1.38)	1.03 (0.68, 1.52)	<0.001
SII	2263.32 (1326.86, 3682.69)	1977.62 (1121.81, 3409.83)	0.002
HGB (g/L)	138.00 (124.00, 150.00)	135.00 (124.00, 146.25)	0.165
PCT (ng/mL)	0.37 (0.10, 3.83)	0.09 (0.04, 0.41)	<0.001
Fecalith discovered on CT (*n* (%))			
Yes	39 (6.1%)	119 (8.1%)	0.102
No	602 (93.9%)	1346 (91.9%)	
Postoperative pathological classification (*n* (%))			
Simple inflamed appendicitis	8 (1.2%)	47 (3.2%)	<0.001
Suppurative appendicitis	197 (30.7%)	708 (48.4%)	
Gangrenous appendicitis with/ without perforation	412 (64.3%)	619 (42.3%)	
Acute exacerbation of chronic appendicitis	21 (3.3%)	85 (5.8%)	
LOS (days)	7 (6, 10)	5 (4, 7)	<0.001
Total hospitalization costs (¥)	20563.91 (17317.58, 25828.46)	16055.75 (13708.90, 18901.77)	<0.001

CHD: coronary heart disease; COPD: chronic obstructive pulmonary disease; SII: systemic immune-inflammation index; LOS: length of stay; CT: computed tomography; Plt: platelet; Neu: neutrophil; Lym: lymphocyte; HGB: haemoglobin; PCT: procalcitonin.

While Plt and Neu counts did not differ significantly between the two groups, a significant variation in Lym resulted in a higher SII in the drainage tube group (median 2263.32 (IQR 1326.86–3682.69)) than that in the non-drainage tube group (median 1977.62 (IQR 1121.81–3409.83); *p* = 0.002). PCT levels exhibited significant differences between the two groups (*p* < 0.001). The LOS for patients who underwent drainage tube placement during surgery (median 7 (IQR 6–10) days) was longer than that for patients without drainage tubes (median 5 (IQR 4–7) days). Similarly, the total hospitalization costs for patients with drainage tubes (median ¥ 20563.91 (IQR ¥ 17317.58–25828.46)) were significantly higher than those for patients without drainage tubes (median ¥ 16055.75 (IQR ¥ 13708.90–18901.77); *p* < 0.001).

### 3.4. Intragroup and intergroup differences between suppurative appendicitis and gangrenous appendicitis

Given the variations in the proportion of patients with drainage tubes placement during surgery among different postoperative pathological classifications (Table 3), patients with suppurative appendicitis and gangrenous appendicitis with or without perforation were further identified, and a Chi-square test was conducted. Significant differences in the proportion of patients with or without drainage tubes were observed between these two postoperative pathological groups (*p* < 0.001; Supplementary Table 2). Furthermore, the two pathological groups were subdivided following the placement of drainage tubes during surgery, and statistical analyses of the relevant variables were conducted independently. Supplementary Tables 3 and 4 show the results. Table 4 presents the results obtained after merging the two patient groups for joint analysis.

**Table 4.** Differences between the groups with/without drainage tubes of the combination of suppurative appendicitis and gangrenous appendicitis with or without perforation.

	Drainage tubes were placed during operations	Drainage tubes were not placed during operations	*p* Value
Sex (*n* (%))			
Male	294 (55.8%)	612 (50.4%)	0.039
Female	233 (44.2%)	602 (49.6%)	
Age (y/o)	50.00 (34.00, 64.00)	38.00 (28.00, 55.00)	<0.001
Advanced age (≥68 y/o)	98 (18.6%)	107 (8.8%)	<0.001
Non-advanced age (<68 y/o)	429 (81.4%)	1107 (91.2%)	
Admission time			
1:00–8:00 am	113 (21.4%)	287 (23.7%)	0.308
Other time	414 (78.6%)	925 (76.3%)	
Body temperature (*n* (%))			
Normal	384 (73.8%)	900 (75.1%)	0.013
Low fever	88 (16.9%)	218 (18.2%)	
Moderate fever	34 (6.5%)	72 (6.0%)	
High fever	14 (2.7%)	9 (0.8%)	
Shock (*n* (%))			
Yes	41 (7.9%)	71 (5.9%)	0.13
No	479 (92.1%)	1128 (94.1%)	
Comorbidities (*n* (%))			
Hypertension, diabetes, CHD and COPD	109 (20.7%)	138 (11.4%)	<0.001
Other diseases	383 (72.8%)	685 (56.5%)	
Pregnancy	2 (0.4%)	24 (2.0%)	
Plt (×10^9^ cells/L)	194.00 (154.00, 234.00)	195.00 (156.00, 236.00)	0.974
Neu (×10^9^ cells/L)	10.92 (8.38, 13.79)	11.02 (8.34, 13.72)	0.809
Lym (×10^9^ cells/L)	0.88 (0.59, 1.35)	0.99 (0.67, 1.47)	0.003
SII	2353.78 (1364.97, 3799.18)	2141.31 (1234.92, 3578.98)	0.047
HGB (g/L)	138.00 (124.00, 150.00)	136.00 (124.00, 147.00)	0.116
PCT (ng/mL)	0.37 (0.10, 4.30)	0.10 (0.05, 0.49)	<0.001
Fecalith discovered on CT (*n* (%))			
Yes	33 (6.3%)	91 (7.5%)	0.358
No	494 (93.7%)	1123 (92.5%)	
LOS (days)	7 (6, 10)	5 (4, 7)	<0.001
Total hospitalization costs (¥)	20666.38 (17385.47, 26104.73)	16101.32 (13778.29, 19009.01)	<0.001

CHD: coronary heart disease; COPD: chronic obstructive pulmonary disease; SII: systemic immune-inflammation index; LOS: length of stay; CT: computed tomography; Plt: platelet; Neu: neutrophil; Lym: lymphocyte; HGB: haemoglobin; PCT: procalcitonin.

Among patients with suppurative appendicitis, gangrenous appendicitis with or without perforation, or within the combined group, those who underwent drainage tube placement were older (*p* < 0.001), with a higher proportion being were of advanced age (≥68 y/o). Furthermore, the incidence of comorbidities (hypertension, diabetes, CHD, COPD and other diseases) was higher among patients in the drainage tube group, while a greater proportion of pregnant patients had not undergone drainage tube placement during surgery (*p* < 0.001). Among blood examination results, only the PCT levels demonstrated consistent elevation in the drainage across all three comparisons (*p* < 0.001). In addition, irrespective of the comparison group, patients who underwent drainage tube placement exhibited significantly longer LOS and higher total hospitalization costs (*p* < 0.001).

### 3.5. Multivariable logistic regression analysis of systemic immune-inflammation index and placement of intraoperative drainage tubes between suppurative appendicitis and gangrenous appendicitis

Since the focus of this study is to provide evidence supporting the determination of intraoperative drainage tube placement, patients diagnosed with suppurative appendicitis and gangrenous drainage tubes appendicitis were further selected for examination. Multivariable logistic regression analysis was conducted using intraoperative drainage tube placement (yes/no) as the dependent variable, while sex, age, body temperature, comorbidities, Lym (×10^9^ cells/L), SII and PCT (ng/mL) were included as covariates (Table 5). Age, body temperature, comorbidities and SII may be considered independent risk factors for intraoperative drainage tube placement. While age constitutes an independent risk factor for intraoperative drainage tube placement (*p* < 0.001; OR = 1.372, 95%CI: 1.190–1.581), no significant correlation was observed between advanced age (≥68 y/o) and the placement of these tubes (*p* = 0.817). Among the classifications of body temperature, only high fever represents an independent risk factor (*p* = 0.003; OR = 3.794, 95%CI: 1.572–9.155). Among the comorbidities classifications, chronic diseases (hypertension, diabetes, CHD and COPD) (*p* < 0.001; OR = 1.853, 95%CI: 1.288–2.666), and other diseases (*p* < 0.001; OR = 1.739, 95%CI: 1.356–2.2296) are identified as independent risk factors. In contrast, pregnancy is not correlated with the placement of intraoperative drainage tubes (*p* = 0.298). Furthermore, the SII constitutes an independent risk factor (*p* = 0.029; OR = 1.124, 95%CI: 1.012–1.248).

**Table 5.** Multivariable logistic regression analysis showing the predictors of whether intraoperative drainage tubes were placed between suppurative appendicitis and gangrenous appendicitis.

	*β*	S.E.	Wald's *χ*^2^	*p* Value	OR	95%CI	
						LL	UL
Sex	−0.184	0.103	3.205	0.073	0.832	0.680	1.018
Age (y/o)	0.316	0.072	19.064	<0.001	1.372	1.190	1.581
Advanced age (≥68 y/o) or not	0.042	0.184	0.053	0.817	1.043	0.727	1.497
Body temperature			8.885	0.031			
Low fever	0.000	0.135	0.000	0.998	1.000	0.768	1.304
Moderate fever	0.069	0.210	0.109	0.741	1.072	0.711	1.616
High fever	1.333	0.449	8.799	0.003	3.794	1.572	9.155
Comorbidities			22.286	<0.001			
Hypertension, diabetes, CHD and COPD	0.617	0.186	11.053	<0.001	1.853	1.288	2.666
Other diseases	0.553	0.127	19.022	<0.001	1.739	1.356	2.229
Pregnancy	−0.780	0.749	1.085	0.298	0.458	0.106	1.989
Lym (×10^9^ cells/L)	0.064	0.050	1.604	0.205	1.066	0.966	1.176
SII	0.117	0.053	4.788	0.029	1.124	1.012	1.248
PCT (ng/mL)	0.037	0.046	0.664	0.415	1.038	0.949	1.136

S.E.: standard error; OR: odds ratio; CI: confidence interval; LL: lower limit; UL: upper limit; CHD: coronary heart disease; COPD: chronic obstructive pulmonary disease; Lym: lymphocyte; SII: systemic immune-inflammation index; PCT: procalcitonin.

## 4. Discussion

In this retrospective study, SII levels demonstrated a positive association with LOS and total hospitalization costs. The treatment methods, pathological classifications, intraoperative drainage tube placement and the severity of acute appendicitis collectively contribute to significant intergroup differences in SII levels.

In 2014, a report described a retrospective study of a primary cohort of 133 patients with hepatocellular carcinoma (HCC). These patients underwent curative resection between 2005 and 2006, alongside a prospective investigation of a validation cohort consisting of 123 patients with HCC undergoing resection. The findings indicate the prognostic value of SII for patients with HCC following surgery. These findings confirms that SII is a novel, independent prognostic predictor for patients undergoing curative resection for HCC, particularly owing to the high recurrence rates observed among those with high SII scores [14]. Subsequently, the SII has been extensively employed in research to predict and assess the prognosis of various tumours.

PCT and CRP are commonly employed to evaluate infection and inflammation. The SII offers several advantages and disadvantages in comparison: (1) SII exhibits higher specificity for complex appendicitis, thereby making it effective for identifying high-risk patients. However, PCT and CRP exhibit greater sensitivity, especially among children. (2) SII provides a reliable assessment of pregnant patients, as it integrates multiple haematological parameters and minimize misdiagnosis due to the physiological leukocytosis. Conversely, the specificity of PCT and CRP may be reduced owing to the inflammatory change associated with pregnancy. (3) The SII demonstrates greater suitability for preoperative evaluation of disease progression. While PCT and CRP levels remain elevated postoperatively–reflecting potential complications–SII provides a more reliable baseline indicator for preoperative status [15,16]. (4) SII is determined using routine blood parameters (Plt, Neu and Lym). This makes it cost-effective and time-efficient. In contrast, the measurement of PCT and CRP require separate serological tests, which are more expensive and time-consuming.

This study is not the first to investigate the relationship between the SII and acute appendicitis. Given the considerable differences between paediatric and adult presentations of the condition, studies involving children were excluded from this analysis. Among the retrieved literature, a retrospective study analysed 161 patients who underwent surgery for acute appendicitis at Trakya University from March 2019 to March 2021. The result shows that SII is directly associated with the presence of complications. Therefore, the SII may function as a predictive marker for preoperative or postoperative complications in acute appendicitis [17]. A cross-sectional retrospective study including 347 patients was conducted between 18 March 2020 and 18 March 2022. The study shows that the SII–a simple, inexpensive and readily accessible parameter–may serve as a valuable adjunct to other clinical findings for diagnosing acute appendicitis in adult patients [18]. Another retrospective study, conducted at a tertiary healthcare centre focused on geriatric patients with suspected acute appendicitis. The result shows that SII exhibits high diagnostic accuracy in differentiating acute appendicitis from non-appendicitis cases among geriatric patients. SII may represent a valuable biomarker for the prompt and accurate diagnosis of acute appendicitis in elderly patients presenting with atypical clinical features [15]. A third retrospective study reviewed data of 441 patients who underwent surgery for acute appendicitis between January 2021 and December 2023, showing that the SII constitutes an effective, reliable and clinically valuable parameter for the preoperative diagnosis of complicated acute appendicitis. This supports surgeons in providing more informed treatment plans and effectively managing patient expectations [19].

The appendix is a tubular prolongation of the cecum. While traditionally considered a vestigial organ, the appendix is now increasingly recognized as an immune structure and microbial reservoir, contributing to mucosal immunity and the maintenance of colonic microbiome stability [20,21]. The appendix exhibits a high density of gut-associated lymphoid tissue and hosts diverse populations of innate immune cells, including natural killer cells and intraepithelial CD8^+^ T cells [22,23]. The tissue also comprises immune inductive sites, including B cell-containing lymphoid follicles with constitutive germinal centres, and immune effector sites characterized based on lamina propria-resident plasma cells producing IgG, IgA and macrophages [21,22,24]. While the appendix serves important physiological functions, prompt surgical intervention remains essential for patients with acute appendicitis. Previous studies show that in-hospital delays are associated with an increased risk of perforation in adults, which correlates with higher complication rates and prolong hospital stays [25,26]. Given the established correlation between the time from symptom onset to appendectomy, pathological severity and complication rates, early surgical intervention is recommended following the capacity of the hospital. These findings align with those of the World Society of Emergency Surgery study group initiative [27,28].

Our analysis reveals that older patients, particularly those around age 45 years, tend to delay seeking medical evaluation for acute appendicitis. This phenomenon indicates that younger individuals may possess stronger awareness, while delays responses among middle-aged adults could be related to limited health education. However, acute appendicitis progresses rapidly from suppuration to gangrene and even perforation, resulting in prolonged hospital stays and increased healthcare costs. Such delays exacerbate the economic burden on the national healthcare system. To address this issue, targeted health education campaigns for individuals around the age of 45 years should be implemented to promote timely medical consultation when experiencing symptoms such as abdominal pain, which may indicate acute appendicitis. Public health promotion and medical science outreach should be intensified, especially among women, to increase awareness of the severity of the disease and promote healthier dietary habits. Furthermore, conservative treatment is not recommended for patients with acute appendicitis during pregnancy.

The placement of abdominal drainage tubes plays an important role in surgical procedures. These tubes facilitate the timely removal of exudate, blood, bile, gastric fluid, intestinal fluid, pancreatic secretions and ascites from the abdominal cavity. Concurrently, surgeons can monitor the volume and characteristics of the drainage fluid to evaluate intra-abdominal conditions and detect postoperative complications early, thereby reducing missed treatment interventions and preventing secondary infections. Consequently, the use of abdominal drains during surgical procedures is routinely practiced by many surgeons. However, the use of abdominal drainage tubes presents certain limitations. These devices can increase patient discomfort and hospitalization costs [29]. The insertion procedure is associated with a risk of abdominal wall hematoma or even drain-site hernia [30,31]. Drainage tubes may exert pressure on intra-abdominal structures, leading to complications such as intestinal obstruction, enteric fistula or intra-abdominal haemorrhage [32,33]. Abdominal drainage tubes can induce abdominal pain, exacerbate psychological stress and extend bed rest, all of which may increase the incidence of hypostatic pneumonia and surgical site infection. The decision to place drainage tubes during surgery is primarily guided by the judgment of surgeons, as no definitive criteria for standardizing this practice. With the growing implementation of enhanced recovery following surgery, an increasing number of experts advocate against the routine drainage tube placement or recommend their early removal. Routine abdominal drainage tube placement is generally not beneficial in standard colorectal surgeries. In contrast, the omission of a drainage tube may reduce the incidence of anastomotic fistulas and wound infections [34].

Based on the findings of this study, we propose a preliminary hypothesis: patients with an SII ≥ 2500 may require intraoperative drainage, while those with an SII ≤ 2000 may not require it. However, these findings are hypothesis-generating, derived from the retrospective study. The clinical applicability of these findings must be confirmed through multi-centre prospective studies. While acute appendicitis during pregnancy typically progresses more rapidly and is more prone to gangrene or perforation than those of other forms of acute appendicitis, >90% of pregnant patients in this study did not undergo abdominal drainage during surgery. In addition, abdominal drainage tubes may stimulate the uterus and foetus, potentially increasing the risk of postoperative complications and adversely affecting prognosis. Therefore, it can be inferred that even among high-risk patients, intraoperative drainage may be unnecessary and unlikely to result in serious adverse outcomes.

This single-centre retrospective study possesses several strengths. First, the sample selection in this study is highly representative, encompassing individuals across all age groups. To our knowledge, this research includes one of the largest sample sizes in China derived from real-world data. Second, covariates such as age, sex, admission time, body temperature, SI, comorbidities, fecalith discovered on CT, intraoperative drainage tube placement and postoperative pathological findings were included in the analysis, thereby enhancing its statistical robustness. Third, as the emergency medical centre for a city of >34 million residents, our hospital possesses extensive experience in managing patients with critical and severe conditions, which may serve as a valuable reference for other cities with similarly sized populations. However, these findings should be interpreted with caution owing to some limitations. As the study is based on a Chinese population, the applicability of these findings to other ethnic groups may be limited. The retrospective study design prevents the establishment of causal relationships, highlighting the need for prospective studies with larger sample sizes to further clarify these findings.

## 5. Conclusions

This study shows that the SII is associated with the prediction of pathological classification at admission, thereby supporting the selection of appropriate treatment strategies and informing decisions regarding intraoperative drainage tube placement. However, further multicentre retrospective and large-scale prospective studies are required to validate these findings. From a health economics perspective, the SII may aid surgeons in identifying target populations for public health education and serve as a reference indicator for patients in whom drainage tube placement may be unnecessary, thereby reducing LOS and minimizing avoidable medical costs.

## Supplementary Material

Supplemental Material

## Data Availability

The authors confirm that the data supporting the findings of this study are available within the article and its supplementary materials. Further inquiries can be directed to the corresponding author, Beige Zong.
