# Supplementary material for: Clinical significance of the systemic immune-inflammation index in relation to pathology, treatment and outcomes in acute appendicitis: a retrospective study
Source: Ann Med. 2025 Nov 18;57(1):2585543. doi: 10.1080/07853890.2025.2585543 (PMC12628667; doi:10.1080/07853890.2025.2585543)
Supplement: Supplemental Material [file IANN_A_2585543_SM3683.docx]

**Supplementary Table 1.** Pairwise comparisons between the groups of postoperative pathological classification.

| Groups | | *P* value |
| --- | --- | --- |
| Age (y/o) | |  |
| Simple inflamed appendicitis | Suppurative appendicitis | ＜0.001 |
| Simple inflamed appendicitis | Gangrenous appendicitis with or without perforation | ＜0.001 |
| Simple inflamed appendicitis | Acute exacerbation of chronic appendicitis | ＜0.001 |
| Suppurative appendicitis | Gangrenous appendicitis with or without perforation | ＜0.001 |
| Suppurative appendicitis | Acute exacerbation of chronic appendicitis | 0.152 |
| Gangrenous appendicitis with or without perforation | Acute exacerbation of chronic appendicitis | 0.562 |
| Plt (×10^9^ cells/L) | |  |
| Simple inflamed appendicitis | Suppurative appendicitis | 0.840 |
| Simple inflamed appendicitis | Gangrenous appendicitis with or without perforation | 0.100 |
| Simple inflamed appendicitis | Acute exacerbation of chronic appendicitis | 0.803 |
| Suppurative appendicitis | Gangrenous appendicitis with or without perforation | ＜0.001 |
| Suppurative appendicitis | Acute exacerbation of chronic appendicitis | 0.510 |
| Gangrenous appendicitis with or without perforation | Acute exacerbation of chronic appendicitis | 0.011 |
| Neu (×10^9^ cells/L) | |  |
| Simple inflamed appendicitis | Suppurative appendicitis | ＜0.001 |
| Simple inflamed appendicitis | Gangrenous appendicitis with or without perforation | ＜0.001 |
| Simple inflamed appendicitis | Acute exacerbation of chronic appendicitis | 0.799 |
| Suppurative appendicitis | Gangrenous appendicitis with or without perforation | ＜0.001 |
| Suppurative appendicitis | Acute exacerbation of chronic appendicitis | ＜0.001 |
| Gangrenous appendicitis with or without perforation | Acute exacerbation of chronic appendicitis | ＜0.001 |
| Lym (×10^9^ cells/L) | |  |
| Simple inflamed appendicitis | Suppurative appendicitis | ＜0.001 |
| Simple inflamed appendicitis | Gangrenous appendicitis with or without perforation | ＜0.001 |
| Simple inflamed appendicitis | Acute exacerbation of chronic appendicitis | 0.625 |
| Suppurative appendicitis | Gangrenous appendicitis with or without perforation | ＜0.001 |
| Suppurative appendicitis | Acute exacerbation of chronic appendicitis | ＜0.001 |
| Gangrenous appendicitis with or without perforation | Acute exacerbation of chronic appendicitis | ＜0.001 |
| SII | |  |
| Simple inflamed appendicitis | Suppurative appendicitis | ＜0.001 |
| Simple inflamed appendicitis | Gangrenous appendicitis with or without perforation | ＜0.001 |
| Simple inflamed appendicitis | Acute exacerbation of chronic appendicitis | 0.843 |
| Suppurative appendicitis | Gangrenous appendicitis with or without perforation | ＜0.001 |
| Suppurative appendicitis | Acute exacerbation of chronic appendicitis | ＜0.001 |
| Gangrenous appendicitis with or without perforation | Acute exacerbation of chronic appendicitis | ＜0.001 |
| PCT (ng/ml) | |  |
| Simple inflamed appendicitis | Suppurative appendicitis | 0.005 |
| Simple inflamed appendicitis | Gangrenous appendicitis with or without perforation | ＜0.001 |
| Simple inflamed appendicitis | Acute exacerbation of chronic appendicitis | 0.012 |
| Suppurative appendicitis | Gangrenous appendicitis with or without perforation | ＜0.001 |
| Suppurative appendicitis | Acute exacerbation of chronic appendicitis | 0.660 |
| Gangrenous appendicitis with or without perforation | Acute exacerbation of chronic appendicitis | 0.017 |
| LOS (days) | |  |
| Simple inflamed appendicitis | Suppurative appendicitis | 0.350 |
| Simple inflamed appendicitis | Gangrenous appendicitis with or without perforation | ＜0.001 |
| Simple inflamed appendicitis | Acute exacerbation of chronic appendicitis | 0.002 |
| Suppurative appendicitis | Gangrenous appendicitis with or without perforation | ＜0.001 |
| Suppurative appendicitis | Acute exacerbation of chronic appendicitis | 0.025 |
| Gangrenous appendicitis with or without perforation | Acute exacerbation of chronic appendicitis | 0.036 |
| Total hospitalization costs (￥) | |  |
| Simple inflamed appendicitis | Suppurative appendicitis | ＜0.001 |
| Simple inflamed appendicitis | Gangrenous appendicitis with or without perforation | ＜0.001 |
| Simple inflamed appendicitis | Acute exacerbation of chronic appendicitis | 0.004 |
| Suppurative appendicitis | Gangrenous appendicitis with or without perforation | ＜0.001 |
| Suppurative appendicitis | Acute exacerbation of chronic appendicitis | 0.827 |
| Gangrenous appendicitis with or without perforation | Acute exacerbation of chronic appendicitis | ＜0.001 |

Plt: Platelet; Neu: Neutrophil; Lym: Lymphocyte; PCT: Procalcitonin.

**Supplementary Table 2.** The result of chi-square test for the patients with suppurative appendicitis or gangrenous appendicitis with or without perforation.

| Group [n (%)] | Suppurative appendicitis | Gangrenous appendicitis with or without perforation |
| --- | --- | --- |
| Drainage tubes were placed during operations | 170 (20.6%) | 357 (39.1%) |
| Drainage tubes were not placed during operations | 657 (79.4%) | 556 (60.9%) |
| χ^2^ | 70.687 | |
| *P* value | ＜0.001 | |

**Supplementary Table 3.** Differences between the groups with or without drainage tubes of suppurative appendicitis.

|  | Drainage tubes were placed during operations | Drainage tubes were not placed during operations | *P* value |
| --- | --- | --- | --- |
| Gender [n (%)] |  |  |  |
| Male | 93 (54.7%) | 321 (48.9%) | 0.174 |
| Female | 77 (45.3%) | 336 (51.1%) |  |
| Age (y/o) | 47.00 (32.00, 62.25) | 37.00 (28.00, 52.00) | ＜0.001 |
| Advanced age (≥68 y/o) | 24 (14.1%) | 51 (7.8%) | 0.010 |
| Non-advanced age (＜68 y/o) | 146 (85.9%) | 606 (92.2%) |  |
| Admission time |  |  |  |
| 1:00 a.m.～8:00 a.m. | 44 (25.9%) | 155 (23.7%) | 0.547 |
| Other time | 126 (74.1%) | 500 (76.3%) |  |
| Body temperature [n (%)] |  |  |  |
| Normal | 138 (81.2%) | 528 (81.7%) | 0.555 |
| Low fever | 24 (14.1%) | 92 (14.2%) |  |
| Moderate fever | 6 (3.5%) | 24 (3.7%) |  |
| High fever | 2 (1.2%) | 2 (0.3%) |  |
| Shock [n (%)] |  |  |  |
| Yes | 6 (3.5%) | 24 (3.7%) | 0.909 |
| No | 164 (96.5%) | 622 (96.3%) |  |
| Comorbidities [n (%)] |  |  |  |
| Hypertension, diabetes, CHD and COPD | 37 (21.9%) | 66 (10.1%) | ＜0.001 |
| Other diseases | 118 (69.8%) | 354 (54.0%) |  |
| Pregnancy | 0 (0.0%) | 9 (1.4%) |  |
| Plt (×10^9^ cells/L) | 214.00 (164.75, 250.25) | 201.00 (160.50, 241.00) | 0.078 |
| Neu (×10^9^ cells/L) | 10.52 (7.87, 12.88) | 10.64 (7.60, 13.17) | 0.771 |
| Lym (×10^9^ cells/L) | 1.14 (0.70, 1.61) | 1.08 (0.71, 1.55) | 0.634 |
| SII | 1898.27 (1121.14, 3088.70) | 1929.71 (1001.70, 3297.71) | 0.949 |
| HGB (g/L) | 138.00 (125.00, 150.00) | 136.00 (125.00, 147.00) | 0.340 |
| PCT (ng/ml) | 0.16 (0.07, 0.88) | 0.08 (0.04, 0.24) | ＜0.001 |
| Fecalith discovered by CT [n (%)] |  |  |  |
|  |  |  |  |
| Yes | 9 (5.3%) | 42 (6.4%) | 0.596 |
| No | 161 (94.7%) | 615 (93.6%) |  |
| LOS (days) | 7 (5, 9) | 5 (4, 7) | ＜0.001 |
| Total hospitalization costs (￥) | 19081.08 (16135.48, 22960.48) | 15553.25 (13447.52, 18020.89) | ＜0.001 |

Plt: Platelet; Neu: Neutrophil; Lym: Lymphocyte; HGB: Hemoglobin; PCT: Procalcitonin.

**Supplementary Table 4.** Differences between the groups with or without drainage tubes of gangrenous appendicitis with or without perforation.

|  | Drainage tubes were placed during operations | Drainage tubes were not placed during operations | *P* value |
| --- | --- | --- | --- |
| Gender [n (%)] |  |  |  |
| Male | 201 (56.3%) | 291 (52.3%) | 0.241 |
| Female | 156 (43.7%) | 265 (47.7%) |  |
| Age (y/o) | 52.00 (36.00, 65.00) | 40.50 (28.00, 57.75) | ＜0.001 |
| Advanced age (≥68 y/o) | 74 (20.7%) | 56 (10.1%) | ＜0.001 |
| Non-advanced age (＜68 y/o) | 283 (79.3%) | 500 (89.9%) |  |
| Admission time |  |  |  |
| 1:00 a.m.～8:00 a.m. | 69 (19.3%) | 132 (23.7%) | 0.116 |
| Other time | 288 (80.7%) | 424 (76.3%) |  |
| Body temperature [n (%)] |  |  |  |
| Normal | 246 (70.3%) | 371 (67.2%) | 0.064 |
| Low fever | 64 (18.3%) | 126 (22.8%) |  |
| Moderate fever | 28 (8.0%) | 48 (8.7%) |  |
| High fever | 12 (3.4%) | 7 (1.3%) |  |
| Shock [n (%)] |  |  |  |
| Yes | 35 (10.0%) | 47 (8.5%) | 0.449 |
| No | 315 (90.0%) | 505 (91.5%) |  |
| Comorbidities [n (%)] |  |  |  |
| Hypertension, diabetes, CHD and COPD | 72 (20.2%) | 72 (12.9%) | ＜0.001 |
| Other diseases | 265 (74.2%) | 330 (59.4%) |  |
| Pregnancy | 2 (0.6%) | 15 (2.7%) |  |
| Plt (×10^9^ cells/L) | 190.00 (148.50, 227.50) | 189.00 (150.00, 230.00) | 0.957 |
| Neu (×10^9^ cells/L) | 11.24 (8.86, 14.64) | 11.69 (9.07, 14.27) | 0.448 |
| Lym (×10^9^ cells/L) | 0.81 (0.54, 1.24) | 0.90 (0.59, 1.35) | 0.029 |
| SII | 2526.99 (1542.74, 4025.95) | 2374.03 (1434.12, 3795.50) | 0.273 |
| HGB (g/L) | 138.00 (123.50, 150.00) | 135.00 (123.00, 146.00) | 0.163 |
| PCT (ng/ml) | 0.56 (0.13, 6.32) | 0.14 (0.06, 1.48) | ＜0.001 |
| Fecalith discovered by CT [n (%)] |  |  |  |
| Yes | 24 (6.7%) | 49 (8.8%) | 0.256 |
| No | 333 (93.3%) | 507 (91.2%) |  |
| LOS (days) | 8 (6, 10) | 6 (4, 8) | ＜0.001 |
| Total hospitalization costs (￥) | 23975.04 (20332.58, 29425.96) | 18191.16 (15655.19, 22080.04) | ＜0.001 |

Plt: Platelet; Neu: Neutrophil; Lym: Lymphocyte; HGB: Hemoglobin; PCT: Procalcitonin.
